# Prognostic Significance of Tumor Volume in Locally Recurrent Nasopharyngeal Carcinoma Treated with Salvage Intensity-Modulated Radiotherapy

**DOI:** 10.1371/journal.pone.0125351

**Published:** 2015-04-30

**Authors:** WeiWei Xiao, Shuai Liu, YunMing Tian, Ying Guan, ShaoMin Huang, ChengGuang Lin, Chong Zhao, TaiXiang Lu, Fei Han

**Affiliations:** 1 Department of Radiotherapy, SunYat-sen University Cancer Center, State Key Laboratory of Oncology in South China, Collaborative Innovation Center of Cancer Medicine, Guangzhou, Guangdong, China; 2 Department of Radiation Oncology, Hui Zhou Municipal Central Hospital, Huizhou, Guangdong, China; University of California Davis, UNITED STATES

## Abstract

**Introduction:**

To evaluate the prognostic value of gross tumor volume (TV) in patients with locally recurrent, nonmetastatic nasopharyngeal carcinoma.

**Methods:**

Between 2001 and 2012, 291 consecutive patients with locally recurrent, nonmetastatic nasopharyngeal carcinoma underwent salvage IMRT were retrospectively reviewed. The correlations between TV and recurrent T classification were analyzed. Survival analyses were performed. Receiver operating characteristic (ROC) curves were calculated to identify cut-off point of TV. The Akaike information criterion and Harrell’s concordance index (c-index) were utilized to test the prognostic value.

**Results:**

The median TV significantly increased with advancing recurrent T classification (P<0.001). The 5-year overall survival rate was 33.2% for the entire cohort. On multivariate analysis, TV was an independent negative prognostic factor for distant metastasis-free survival (hazard ratio =1.013, P =0.003), overall survival (hazard ratio = 1.015, P<0.001) and toxicity-related death (hazard ratio = 1.014, P<0.001). The 5-year overall survival rates were 63.1% and 20.8% for patients with a TV < 22 cm^3^ and TV ≥22 cm^3^, respectively (P < 0.001). In patient with TV <22 cm^3^, locoregional failure is the leading cause of death. In patients with TV≥22 cm^3^, distant metastasis rate is higher and occurred within short term after local recurrence; meanwhile, radiation-induced injuries became more common and led to half of deaths in this group. The Akaike information criterion and c-index analyses indicated that the predictive ability of recurrent T classification improved when combined with TV.

**Conclusions:**

Our data suggests TV is a significant prognostic factor for predicting the distant metastasis, overall survival and toxicity-related death of patients with locally recurrent, nonmetastatic nasopharyngeal carcinoma after salvage IMRT. TV should be considered when designing personalized salvage treatments for these patients. For patients with bulky local recurrent tumor, radiation may need to be de-emphasized in favor of systemic treatment or best supportive care.

## Introduction

Radiotherapy is the first-line treatment for primary NPC patients [1]. Local recurrence after the first course of radiotherapy is a challenging problem for oncologists. Intensity-modulated radiotherapy (IMRT) is an option for the salvage treatment of locally recurrent NPC patients and could achieve long-term survival for some of these patients. However, locally recurrent NPC is a highly heterogeneous disease, and patient survival after IMRT varies.

The primary tumor volume represents a significant independent prognostic factor in most malignant tumors, including primary NPC, in both the two-dimensional radiotherapy [2–4] and the IMRT eras [5–7]. The prognostic value of tumor volume in recurrent NPC patients remains far from clear.

To address this issue, we conducted this retrospective study of patients to investigate the significance of tumor volume on survival outcome of locally recurrent, nonmetastatic NPC who were treated with salvage IMRT, to determine the value of the tumor volume compared with established prognostic staging systems and to improve the personalized treatment of NPC patients with locally recurrent, nonmetastatic disease.

## Patients and Methods

### Ethics statement

This study was approved by our Institutional Review Boards (IRBs) for Cancer Center, Sun Yat-sen University. Written informed consents were obtained from all the patients in accordance with the regulations of IRBs.

### Patient characteristics

We retrospectively reviewed the records of 291 patients with locally recurrent, non-metastatic NPC were treated with salvage IMRT at our center between January 2001 and April 2012. The inclusion criteria were as follows: (1) patients between 20–80 years of age; (2) histopathologically or radiologically diagnosed as having locally recurrent NPC; (3) IMRT were used for salvage treatment. Patients who had distant metastasis were excluded from this study. The characteristics of the 291 patients are presented in Table 1.

**Table 1:** Characteristics of the 291 patients with locally recurrent NPC.

Patient characteristics	No. of patients (%)
Age (years)	
Median	46
Range	21–79
Gender	
Male	225 (77.3)
Female	66 (22.7)
Initial treatment	
Radiotherapy alone	205(70.4)
CCRT±neoadjuvant/adjuvant chemotherapy	75(25.8)
Radiotherapy+ neoadjuvant/adjuvant chemotherapy	11(37.8)
Initial radiotherapy mode	
2D-RT	265(91.1)
3D-CRT	10(3.4)
IMRT	7(2.4)
2D-RT+brachetherapy	8(2.7)
3D-CRT+brachetherapy	1(0.3)
Initial radiotherapy dose	
≤70Gy	179(61.5)
>70Gy	112(38.5)
Recurrence interval (months)	
Median	26
Range	6–265
Histology	
WHO type I	9 (3.1)
WHO type II-III	232 (79.7)
Imaging findings only	50 (17.2)
Recurrent T classification*	
rT1	20 (6.9)
rT2	27 (9.3)
rT3	117 (40.2)
rT4	127 (43.6)
Recurrent N classification*	
rN0	238 (81.8)
rN1	43 (14.8)
rN2	6 (2.1)
rN3	4 (1.4)
Recurrent clinical stage*	
rI	15 (5.2)
rII	30 (10.3)
rIII	113 (38.8)
rIVA-B	133 (45.7)
Salvage treatment	
Salvage IMRT alone	45 (15.5)
Salvage IMRT + chemotherapy	246 (84.5)

Abbreviations: CCRT, concurrent chemoradiotherapy

* According to the 7th AJCC/UICC staging system.

### Clinical staging

All of the patients completed a pretreatment evaluation, including a complete patient history, physical examination and hematology and biochemistry profiles. Magnetic resonance imaging (MRI) or computed tomography (CT) of the nasopharynx and neck was performed for the staging evaluations. Chest radiography, abdominal ultrasonography and a whole-body bone scan using single-photon emission computed tomography (ECT) were performed to exclude distant metastasis. Positron emission tomography (PET)-CT was not compulsory but was performed at the physician’s discretion. All of the patients were restaged according to the 7th edition of the International Union against Cancer/American Joint Committee on Cancer (UICC/AJCC) system [8].

### Tumor volume measurement

The gross recurrent tumor volume (TV) was manually outlined in the planning system according to the pretreatment MRI image by a radiation oncologist and then was verified by two additional radiation oncologists who specialize in NPC treatment. If tumor volume was decreased by induction chemotherapy, the tumor targets were contoured according to the post-chemotherapy images. The TV values were calculated using the planning system and the summation-of-area technique, which multiplies the entire area by the image reconstruction interval of 3 mm.

### Intensity-modulated radiotherapy

All of the patients were immobilized in the supine position using a head, neck and shoulder thermoplastic mask. Pretreatment contrast-enhanced CT imaging was performed to obtain 3-mm slices from the head to 2 cm below the sternoclavicular joint, and the images were transferred to the CORVUS inverse IMRT planning system (version 3.0; Peacock, 3.0; NOMOS Corp, Sewickley, PA, USA). Target volumes were delineated according to our institution’s treatment protocol, which is in agreement with the International Commission on Radiation Units and Measurements (ICRU) reports 50 and 62. The recurrent gross tumor volumes (rGTV) at the primary site (rGTV-nx) and the neck (rGTV-nd) included the total disease volumes visualized using CT or MRI, and the clinical target volumes (CTVs) were contoured as in our previous reports [9–10]. PTVs were generated for setup variability and internal motion. The organs at risk (OARs) were contoured, and dose constraints to the OARs were limited by the threshold doses as reported previously. The prescribed doses were 60–70 Gy to the GTV and 50–54 Gy to the CTV in 27–35 fractions. All of the patients received IMRT with 6-MV X-rays generated using a Clinac-600C linear accelerator (Varian Medical Systems, Palo Alto, CA, USA). All of the patients completed the planned IMRT courses.

### Chemotherapy

Cisplatin-based induction or concurrent chemotherapy was administered to 246 patients with rIII-IV disease and/or bulk gross tumors. The cohort included 120 patients treated with concurrent chemoradiotherapy, 104 patients treated with induction chemotherapy followed by radiotherapy, 16 patients treated with induction and concurrent chemotherapy, and 6 patients treated with radiotherapy followed by adjuvant chemotherapy.

### Patient follow-up and statistical analysis

The duration of follow-up was calculated from the diagnosis of recurrence to either the day of death or the day of the last follow-up. Patients were seen every 3 months during the first 2 years, and every 6 months thereafter until death. The end points (time to the first defining event) which were assessed included overall survival (OS), local failure-free survival (LFFS) and distant metastasis-free survival (DMFS) and toxicity-related death (TRD).

The Kruskal-Wallis test was used to compare the differences in the TV among patients with various stage diseases. Receiver operating characteristic (ROC) curve analysis was used to evaluate the different TV cut-off points.

Actuarial rates were calculated using the Kaplan-Meier method, and differences were compared using the log-rank test. Univariate and multivariate analyses using a Cox proportional hazards model were utilized to test the independent significance of different factors by backward elimination. Host factors (age, sex, recurrence interval and WHO histological grade), tumor factors (recurrent T and recurrent N classification, recurrent clinical stage and TV), treatment factors (initial radiotherapy dose and chemotherapy) were included as covariates in all of the analyses.

The prognostic stratification of survival by recurrent T classification and TV was evaluated using the Akaike information criterion (AIC) [11] and Harrell’s concordance index (c-index) [12]. The AIC was analyzed using the Cox proportional hazards regression model. The optimal model—the simplest effective model with the smallest information loss when predicting outcome—gives the lowest AIC value. Harrell’s c-index was also calculated as a measure of the predictive accuracy of survival outcome; a c-index of 0.5 indicates accuracy similar to random guessing, and that of 1.0 indicates 100% predictive accuracy.

All of the analyses were performed using the SPSS software, version 16.0 (SPSS, Chicago, IL, USA) and R version 3.0.3 (www.r-project.org). The criterion for statistical significance was set at P = 0.05 and P values were based on two-sided tests.

## Results

### Treatment outcome and death reason

The median follow-up duration for the entire cohort was 29 months (range: 3.1–146 months).

The common severe late normal tissue effects observed before re-irradiation included ulcer or necrosis of the nasopharyngeal mucosa with the incidence rate of 10.7% (31/291), trismus with the incidence rate of 8.2% (24/291), temporal lobe necrosis with the incidence rate of 4.5% (13/291), cranial nerve palsy with the incidence rate of 3.1% (9/291), hearing deficit with the incidence rate of 3.1% (9/291), and vision deficit with the incidence rate of 0.7% (2/291).

After reirradiation, 98 patients (33.7%) had ulcer or necrosis of the nasopharyngeal mucosa, 88 patients (30.2%) with trismus, 78 patients (30%) with temporal lobe necrosis (TLN), 50 patients (17.2%) with massive hemorrhage, 70 patients (24%) with hearing deficit, 56 patients (19.2%) with severe headache, 16 patients (5.5%) with difficulty in feeding. 15 patients (5.1%) with difficulty in speaking, 13 patients (4.5%) with vision deficit.

**Fig 1:**
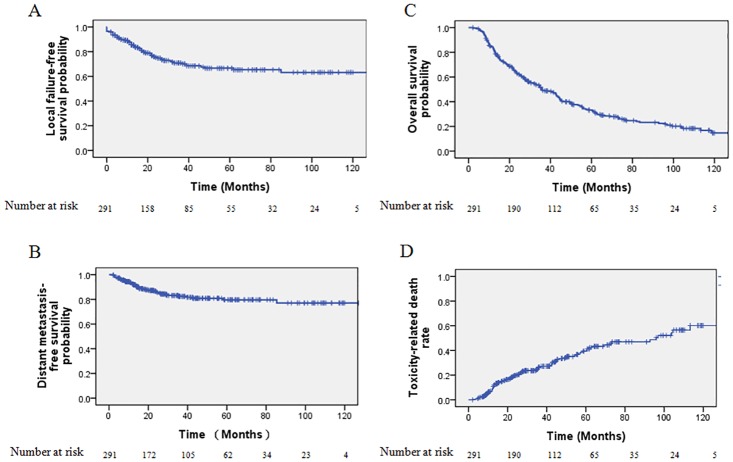
Kaplan-Meier survival curves of the local failure-free survival (A), distant metastasis-free survival (B), overall survival (C) and toxicity-related death (D) of all 291 locally recurrent nasopharyngeal carcinoma patients.

A total of 73/291 (25.1%) patients developed local failure, 10/291 (3.4%) patients developed regional failure, 2/291(0.7%) patients developed local and regional failure and 44/291 (15.1%) developed distant metastases. The 5-year LFFS rate and DMFS was 66.6% and 79.6% respectively (Fig 1A and 1B).

A total of 201/291 (69.1%) patients died. The 5-year OS rate for the entire cohort was 33.2% (Fig 1C). The median OS period was 36 months (95% confidence interval [CI], 28.6–43.4 months). Of the 201 patients who died, 56/291 (19.2%) died because of locoregional failure, 29/291 (10.0%) died because of distant failure and 10/291 (3.4%) died because of both locoregional failure and distant failure. In addition, 94/291 (32.3%) deaths were due to radiation-induced injuries, including 57/291 (19.6%) from mucosa necrosis or massive hemorrhage, 12/291 (4.1%) from radiation encephalopathy and 11 (3.8%) from feeding difficulty and 14/291 (4.8%) from other radiation-related injuries. The 5-year TRD rate for the entire cohort was 39.5% (Fig 1D). Other causes responsible for 12 deaths included 3/291 (1.0%) cases of internal medical disease, 1/291 (0.3%) case of leukemia, 1/291 (0.3%) case of brain stem infarction, 1/291 (0.3%) case of encephalatrophy and 6/291 (2.1%) unknown causes.

### Characteristics and analysis of TV as a prognosis factor

The median primary tumor volume was 36.22 cm^3^ (range: 0.81–158.89 cm^3^) for all 291 patients, 14.29 cm^3^ (range: 0.81–127.14 cm^3^) for patients with stage rT1 tumors, 15.38 cm^3^ (range: 7.63–38.29 cm^3^) for patients with stage rT2 tumors, 31.13 cm^3^ (range: 6.97–158.89 cm^3^) for patients with stage rT3 tumors and 48.90 cm^3^ (range: 8.02–146.25 cm^3^) for patients with stage rT4 tumors. Although overlaps were observed in the TV of patients at different recurrent clinical stages, the median TV significantly increased with advanced recurrent rT classification (χ^2^ = 79.905; P < 0.001).

On univariate analyses, TV was a significant prognostic factor with a hazard ratio (HR) of 1.013, 1.015 and 1.014 for DMFS, OS and TRD (P = 0.003, P <0.001, P <0.001), but not LFFS (P = 0.194); the corresponding 95% confidence interval (CI) of HR was 1.004–1.022 for DMFS, 1.011–1.019 for OS and 1.008–1.020 for TRD. In the multivariate analysis using recurrence interval, age, gender (female vs. male), World Health Organization histological grade (Type II vs. Type I), recurrent T classification (rT3-4 vs. rT1-2), recurrent N classification (rN1-3 vs. rN0), and chemotherapy (with vs. without) as a covariate, the TV remained an independent prognostic factor for DMFS (HR = 1.013, 95% CI 1.004–1.022; P = 0.003; Table 2), OS (HR = 1.015, 95% CI 1.011–1.020; P<0.001; Table 2) and TRD (HR = 1.014, 95% CI 1.007–1.021; P<0.001; Table 2).

**Table 2:** Multivariate analysis of prognostic factors in the 291 patients with locally recurrent NPC receiving salvage IMRT.

Endpoint	Variable	HR	95% CI	P value
LFFS	Recurrent T classification* (rT3-4vs. r1-2)	3.837	1.543–9.542	0.004
DMFS	TV	1.013	1.004–1.022	0.003
OS	TV	1.015	1.011–1.020	<0.001
	Age	1.025	1.012–1.038	<0.001
	Recurrent T classification* (rT3-4vs. r1-2)	1.916	1.206–3.042	0.006
	Recurrence interval	0.994	0.990–0.998	0.003
TRD	TV	1.014	1.007–1.021	<0.001
	Age	1.026	1.007–1.045	0.006
	Recurrence interval	0.994	0.989–1.000	0.048

Abbreviations: TV, gross recurrent tumor volume; HR, hazard ratio; CI, confidence interval; LFFS, local failure-free survival; DMFS, distant metastasis-free survival; OS, overall survival; TRD, toxicity-related death.

* According to the 7th AJCC/UICC staging system. The following parameters were included in the Cox proportional hazards model by backward elimination: recurrence interval as a continuous variable, age as a continuous variable, gender (female vs. male), World Health Organization (WHO) histological grade (Type II vs. Type I), recurrent T classification (rT3-4 vs. rT1-2), recurrent N classification (rN1-3 vs. rN0), initial radiotherapy dose (>70Gy vs. ≤ 70Gy), use of chemotherapy (with vs. without) and TV as a continuous variable.

### Prognostic significance of the TV cut-off

**Fig 2:**
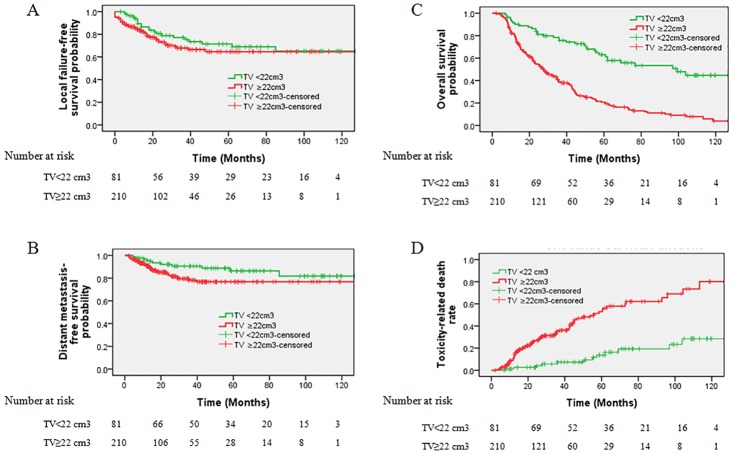
Stratified analyses of all 291 locally recurrent nasopharyngeal carcinoma patients using the gross recurrent tumor volume (TV) cut-off point (<22 cm^3^ vs. ≥22 cm^3^) to compare overall survival (A), local failure-free survival (B), distant metastasis-free survival(C) and toxicity-related death (D).

The cut-off of TV for OS was 22 cm^3^ (sensitivity 82.1%, specificity 50%) and we selected 22 cm^3^ to classify patients into subgroups for survival analysis. The 81 patient with a TV <22 cm^3^ had a significant better OS rate than those 210 patients with a TV ≥22 cm^3^ (63.1% vs. 20.8%, χ2 = 46.529, P<0.001, Fig 2A). The death reasons of the two groups were listed in Table 3. Tumor progression, radiation-induced injuries and other causes led to death in 21(26%), 13 (16.0%) and 2(2%) patients in the TV < 22 cm^3^ group, in contrast to 74 (35%), 81 (39%) and 10 (4%) in the TV ≥22 cm^3^ group.

**Table 3:** Causes of death in the 291 patients and two subgroups.

Causes of death		No. of deaths		
		All (201/291)	TV<22cm^3^ (36/81)	TV≥22 cm^3^ (165/210)
Tumor progression				
(n = 95)	Locoregional failure	56 (28%)	12 (15%)	44 (21%)
	Distant failure	29 (10%)	5 (6%)	24 (11%)
	Locoregional failure and distant failure	10 (4%)	4 (5%)	6 (3%)
Radiation-induced injuries				
(n = 93)	Mucosa necrosis or massive hemorrhage	57 (20%)	8 (10%)	49 (23%)
	Radiation encephalopathy	12 (4%)	1 (1%)	11 (5%)
	Feeding difficulty	11 (3.8%)	1 (1%)	10 (4.8%)
	Other radiation-related injuries	14 (4.8%)	3 (3.7%)	11 (5.2%)
Other causes				
(n = 12)	Internal medical disease	3 (1%)	0	3 (1%)
	Leukemia	1 (0.5%)	1 (1%)	0
	Brain stem infarction	1 (0.5%)	0	1 (0.5%)
	Encephalatrophy	1 (0.5%)	0	1 (0.5%)
	Unknown causes	6 (2%)	1 (1%)	5 (2%)

Abbreviations: No, number; TV, gross recurrent tumor volume; CI, confidence interval

Tumor progression included locoregional failure and distant metastasis. No significant difference was found between patients with a TV <22 cm^3^ and TV ≥22 cm^3^ concerning the 5-year LFFS (71.4% vs. 64.6%, χ2 = 1.206, P = 0.272, Fig 2B). The corresponding DMFS was 86.3% compared with 76.7% (χ2 = 3.161, P = 0.075, Fig 2C), which’s difference is marginally significant. Moreover, 7 (70%) of the recorded distant metastases in 10 patients occurred within 3 years and 3 (30%) after 3 years in the group of TV <22 cm^3^. In contrast, 33(97.1%) of the recorded distant metastases in 34 patients occurred within 3 years after diagnosis in the group of TV ≥22 cm^3^, and 1 occurred 40 months after diagnosis.

In patient with TV <22 cm^3^, tumor progression, especially locoregional failure, is the leading cause of death. In contrast, in patients with TV ≥22 cm^3^, radiation-induced injuries including mucosa necrosis or massive hemorrhage, radiation encephalopathy and other radiation-related injuries became more common and led to half of deaths in this group. Patient with a TV <22 cm^3^ had a significant lower TRD rate than those patients with a TV ≥22 cm^3^ (16.3% vs. 54.6%, χ2 = 31.347, P<0.001, Fig 2D).

### Prognostic validity of adding TV to the recurrent T classification

The AIC and c-index were 1990.0 and 0.669, respectively, for the rT classification alone, and 1944.3 and 0.677, respectively, when TV (< 22 cm^3^ and ≥22 cm^3^) was added to the rT classification. The results revealed that, in predicting overall survival, the addition of TV to rT classification was superior to rT classification alone.

## Discussion

The current study is to assess the prognostic value of the TV in salvage IMRT-treated patients with locally recurrent NPC and to determine the correlation with rT classification. Our analyses provided two principle findings. First, the TV provides valuable prognostic information for IMRT-treated patients with locally recurrent NPC, specifically for DMFS, OS and TRD, but not LFFS. Second, the combination of the TV and rT classification may help classifying these patients into subgroups with significantly different prognosis, likely aiding patient selection.

### TV is prognostic of DMFS, OS and TRD, but not LFFS for locally recurrent NPC treated with salvage IMRT

A larger tumor volume is associated with an increased number of tumor cells, greater radioresistance, increased tumor hypoxia and a higher tendency for distant metastasis [17–19]. The significance of the primary tumor volume in the treatment outcome of primary NPC patients has been previously investigated, and the data indicated an adverse correlation between the TV and local control, distant control and OS [5, 7, 13].

We previously have reported two retrospective studies of 239 local recurrent NPC patients [9] and 251 patients [20] who were re-irradiated with IMRT in our Cancer Center, which emphasized on reporting the treatment outcomes and establishing a prognostic model, respectively. In those two studies, we both found TV was a significant prognostic factor for OS of these patients, in line with the current study, but the prognostic value of TV in other survival endpoints was not investigated. In contrast to primary NPC, TV losses its prognostic significance for the local control of locally recurrent NPC patients after salvage IMRT. The biology of the recurrent tumor is different from the primary tumor characterized by more radioresistance and hypoxia. Whether high dose of irradiation would be helpful for these tumors remains unanswered. Even more difficult is, a substantial portion of patients died from radiation toxicity, which is similar to reports from other center [21]. TV is significantly prognostic of TRD, which means extreme high dose reirradiation may cause more TRD, especially in patients with large TV. Ideal total dose and fraction scheme is not determined for locally recurrent NPC yet, especially when distinguishing the true reason of ulcer or necrosis of the nasopharyngeal mucosa and massive hemorrhage remains difficult. It’s reasonable to design distinct irradiation regimen for patients with different TV, trying to balance maximizing local control and minimizing severe toxicities. Conquer of radioresistance and further increase of local control may still need other treatment modalities [22].

Failure of distant control is another important reason of failure after salvage IMRT for locally recurrent NPC patients. For locally recurrent NPC patients, a larger tumor volume is also supposed to be associated with a higher probability of distant metastasis. In our study, the TV proved to be an independent prognostic factor for DMFS, which is similar to the results from primary NPC series [5, 7]. More effective treatment is needed aiming to decrease the distant metastasis rate for patients with larger TV.

The risk of death was estimated to increase by 1.5% for every 1 cm^3^ increase in the locally recurrent TV, contributing by higher rates of TRD and distant metastasis. Individualized radiotherapy scheme, different chemoradiotherapy combination model, favorable sequence of surgery and radiotherapy, other anti-tumor agents and best supportive care are all in urgent need for salvaging these patients.

### Selection of TV cut-off points

Both one cut-off point and multiple cut-off points have been used in previous studies of primary NPC patients [5–7, 13, 14]. Cut-off points with optimal sensitivity and specificity should be used in clinical practice and chosen by ROC curve analysis to maximize the Youden Score [15, 16]. Based on the ROC analysis, we selected one cut-off point for the whole cohort that could be conveniently clinically applied and that better stratified the patients into subgroups. Therefore, the TV cut-off value of 22 cm^3^ was selected for predicting adverse effects on survivals. This value is approximate to the cut-off values used in the studies investigating primary NPC reported by Sze W [13] in the two-dimensional conventional radiotherapy era and Guo R in the IMRT era [5].

However, in our previous reports, we selected 38 cm^3^ and 30 cm^3^, which were the median value and a value close to the mean value of TV, respectively. Those two cutoff points were also useful to divide patients into groups of different risk. Selecting a specific cutoff point was only for quantitatively illustrating the prognostic value of TV, rather than an absolute dividing line. More sophisticated prognostic model taking TV as a continuous variable is worth of investigation, as in the Chua DT’s study [23].

### The prognostic value of the tumor volume: patient selection and dose prescription for salvage IMRT

Salvage treatment for locally recurrent NPC patients remains challenging. In this analysis, patients with TV <22 cm^3^ had much better long-term survival outcome than those patients with TV ≥22 cm^3^. Tumor progression and radiation-induced injuries are both predominant reasons of death in this kind of patients.

The ideal goal of salvage IMRT is to optimize the total radiation dose for each individual patient to achieve the maximal chance of a cure with the fewest complications, which is more complicated and difficult for locally recurrent NPC than primary tumor. In the TV <22 cm^3^ group, locoregional failure is the leading cause of death, implying that our treatment protocols may be sufficient for distant control and generate moderate late toxicities in this group. Increasing local control is the next goal by changing IMRT dose scheme and/or adding radiosensitizers in the IMRT treatment.

However, in patients with TV≥22 cm^3^, radiation-induced injuries, including mucosa necrosis, massive hemorrhage, radiation encephalopathy and others became the leading cause of death (a half) in this group due to larger irradiated volume. Besides, distant metastasis rate is higher and occurred within short term after diagnosis of local recurrence. For patients with bulky recurrent tumor, we reckon whether radiation may need to be de-emphasized by decreasing the radiotherapy dose to an appropriate level will be helpful to prolong the overall survival time, even if with risk of lower local tumor control probability. Meanwhile, putting more emphasis on systemic treatment or best supportive care aiming to decrease distant failure rate and prolong overall survival time may be warranted.

Another objective of the present study was to verify the prognostic value of adding the TV to rT classification. The AIC and c-index results suggest that the prognostic assessment could be improved by combining the current rT classification with the TV, thereby illustrating the limitations of the current rT classification. An improved predictive ability would allow patients with a worse prognosis to receive more appropriate treatment, which is warranted further investigation.

## Conclusions

In summary, this study reported the prognostic value of the TV in locally recurrent NPC patients treated with IMRT. Our data suggest that the TV is a significant factor for predicting the DMFS, OS and TRD rate of patients with locally recurrent, nonmetastatic NPC after salvage IMRT, but not prognostic for LFFS. The risk of death was estimated to increase by 1.5% for every 1 cm^3^ increase in locally recurrent TV. The TV should be considered when designing personalized salvage treatments for patients with locally recurrent NPC. For patients with bulky recurrent tumor, radiation may need to be de-emphasized in favor of systemic treatment or best supportive care.

## Supporting Information

S1 Fig Good response of locally recurrent tumor after neoadjuvant chemotherapy (E, F, G, H) compared with recurrence (A, B, C, D). (TIF)

S1 Table Major late toxicities of patients with different initial radiation dose. (DOCX)

S1 File Original data of this study. (XLS)

## References

[1] Wei WI, Sham JST. Nasopharyngeal carcinoma. Lancet. 2005; 365:2041–2054. 15950718 10.1016/S0140-6736(05)66698-6

[2] Chang CC, Chen MK, Liu MT, Wu HK, Hwang KL. Effect of primary tumour volumes in early T-stage nasopharyngeal carcinoma. J Otolaryngol. 2003; 32:87–92. 12866592 10.2310/7070.2003.37253

[3] Chen MK, Chen TH, Liu JP, Chang CC, Chie WC. Better prediction of prognosis for patients with nasopharyngeal carcinoma using primary tumor volume. Cancer 2004; 100:2160–2166. 15139059 10.1002/cncr.20210

[4] Lee CC, Chu ST, Ho HC, Lee CC, Hung SK. Primary tumor volume calculation as a predictive factor of prognosis in nasopharyngeal carcinoma. Acta Otolaryngol. 2008; 128:93–97. 17851945 10.1080/00016480701361921

[5] Guo R, Sun Y, Yu XL, Yin WJ, Li WF, Chen YY, et al Is primary tumor volume still a prognostic factor in intensity modulated radiation therapy for nasopharyngeal carcinoma? Radiother Oncol. 2012; 104:294–299. 10.1016/j.radonc.2012.09.001 22998947

[6] Wu Z, Zeng RF, Su Y, Gu MF, Huang SM. Prognostic significance of tumor volume in patients with nasopharyngeal carcinoma undergoing intensity-modulated radiation therapy. Head Neck. 2013; 35:689–694. 10.1002/hed.23010 22715047

[7] Chen C, Fei Z, Pan J, Bai P, Chen L. Significance of primary tumor volume and T-stage on prognosis in nasopharyngeal carcinoma treated with intensity-modulated radiation therapy. Jpn J Clin Oncol. 2011; 41:537–542. 10.1093/jjco/hyq242 21242183

[8] Edge SB, Compton CC, Edge SB, Fritz AG, Greene FL, Trotti A. AJCC cancer staging manual. 7th ed Philadelphia (PA): Lippincott-Raven; 2009.

[9] Han F, Zhao C, Huang SM, Lu LX, Huang Y, Deng XW, et al Long-term outcomes and prognostic factors of re-irradiation for locally recurrent nasopharyngeal carcinoma using intensity-modulated radiotherapy. Clin Oncol (R Coll Radiol). 2012; 24:569–576. 10.1016/j.clon.2011.11.010 22209574

[10] Hua YJ, Han F, Lu LX, Mai HQ, Guo X, Hong MY, et al Long-term treatment outcome of recurrent nasopharyngeal carcinoma treated with salvage intensity modulated radiotherapy. Eur J Cancer. 2012; 48:3422–3428. 10.1016/j.ejca.2012.06.016 22835782

[11] Akaike H. Information theory and an extension of the maximum likelihood principle. Budapest: Akademia Kiado; 1973.

[12] Harrell FE Jr, Lee KL, Mark DB. Multivariable prognostic models: issues in developing models, evaluating assumptions and adequacy, and measuring and reducing errors. Stat Med. 1996; 15:361–387. 8668867 10.1002/(SICI)1097-0258(19960229)15:4<361::AID-SIM168>3.0.CO;2-4

[13] Sze W, Lee A, Yau T, Yeung RM, Lau KY, Leung SK, et al Primary tumor volume of nasopharyngeal carcinoma: prognostic significance for local control. Int J Radiat Oncol Biol Phys. 2004; 59:21–22. 15093895 10.1016/j.ijrobp.2003.10.027

[14] Sarisahin M, Cila A, Ozyar E, Yıldız F, Turen S. Prognostic significance of tumor volume in nasopharyngeal carcinoma. Auris Nasus Larynx. 2011; 38:250–254. 10.1016/j.anl.2010.09.002 20970934

[15] Zweig MH, Campbell G. Receiver-operating characteristic (ROC) plots: a fundamental evaluation tool in clinical medicine. Clin Chem 1993; 39:561–577. 8472349

[16] Yu KJ, Hsu WL, Pfeiffer RM, Chiang CJ, Wang CP, Lou PJ, et al Prognostic utility of anti-EBV antibody testing for defining NPC risk among individuals from high-risk NPC families. Clin Cancer Res. 2011; 17:1906–1914. 10.1158/1078-0432.CCR-10-1681 21447725 PMC3268357

[17] De JK, Merlo FM, Kavanagh MC, Fyles AW, Hedley D, Hill RP. Heterogeneity of tumor oxygenation: relationship to tumor necrosis, tumor size, and metastasis. Int J Radiat Oncol Biol Phys. 1998; 42:717–721. 9845083 10.1016/s0360-3016(98)00323-x

[18] Hill RP, De Jaeger K, Jang A, Cairns R. pH, hypoxia and metastasis. Novartis Found Symp. 2001; 240:154–165; discussion 165–168. 11727927 10.1002/0470868716.ch11

[19] Evans SM, Koch CJ. Prognostic significance of tumor oxygenation in humans. Cancer Lett. 2003; 195:1–16. 12767506 10.1016/s0304-3835(03)00012-0

[20] Tian YM, Tian YH, Zeng L, Liu S, Guan Y, Lu TX, et al Prognostic model for survival of local recurrent nasopharyngeal carcinoma with intensity-modulated radiotherapy. Br J Cancer. 2014; 110:297–303. 10.1038/bjc.2013.715 24335924 PMC3899759

[21] Chen HY, Ma XM, Ye M, Hou YL, Xie HY, Bai YR. Effectiveness and toxicities of intensity-modulated radiotherapy for patients with locally recurrent nasopharyngeal carcinoma. PLoS One. 2013; 8:e73918 10.1371/journal.pone.0073918 24040115 PMC3769398

[22] Suárez C, Rodrigo JP, Rinaldo A, Langendijk JA, Shaha AR, Ferlito A. Current treatment options for recurrent nasopharyngeal cancer. Eur Arch Otorhinolaryngol. 2010; 267:1811–1124. 10.1007/s00405-010-1385-x 20865269 PMC2966947

[23] Chua DT, Sham JST, Hung KN, Leung LH, Au GK. Predictive factors of tumor control and survival after radiosurgery of local failures of nasopharyngeal carcinoma. Int J Radiat Oncolo Biol Phys. 2006; 66:1415–1421. 17056191 10.1016/j.ijrobp.2006.07.1364

