# Supplementary figures and images for: Prognostic Significance of Tumor Volume in Locally Recurrent Nasopharyngeal Carcinoma Treated with Salvage Intensity-Modulated Radiotherapy
Source: PLoS One. 2015 Apr 30;10(4):e0125351. doi: 10.1371/journal.pone.0125351 (PMC4416016; doi:10.1371/journal.pone.0125351)

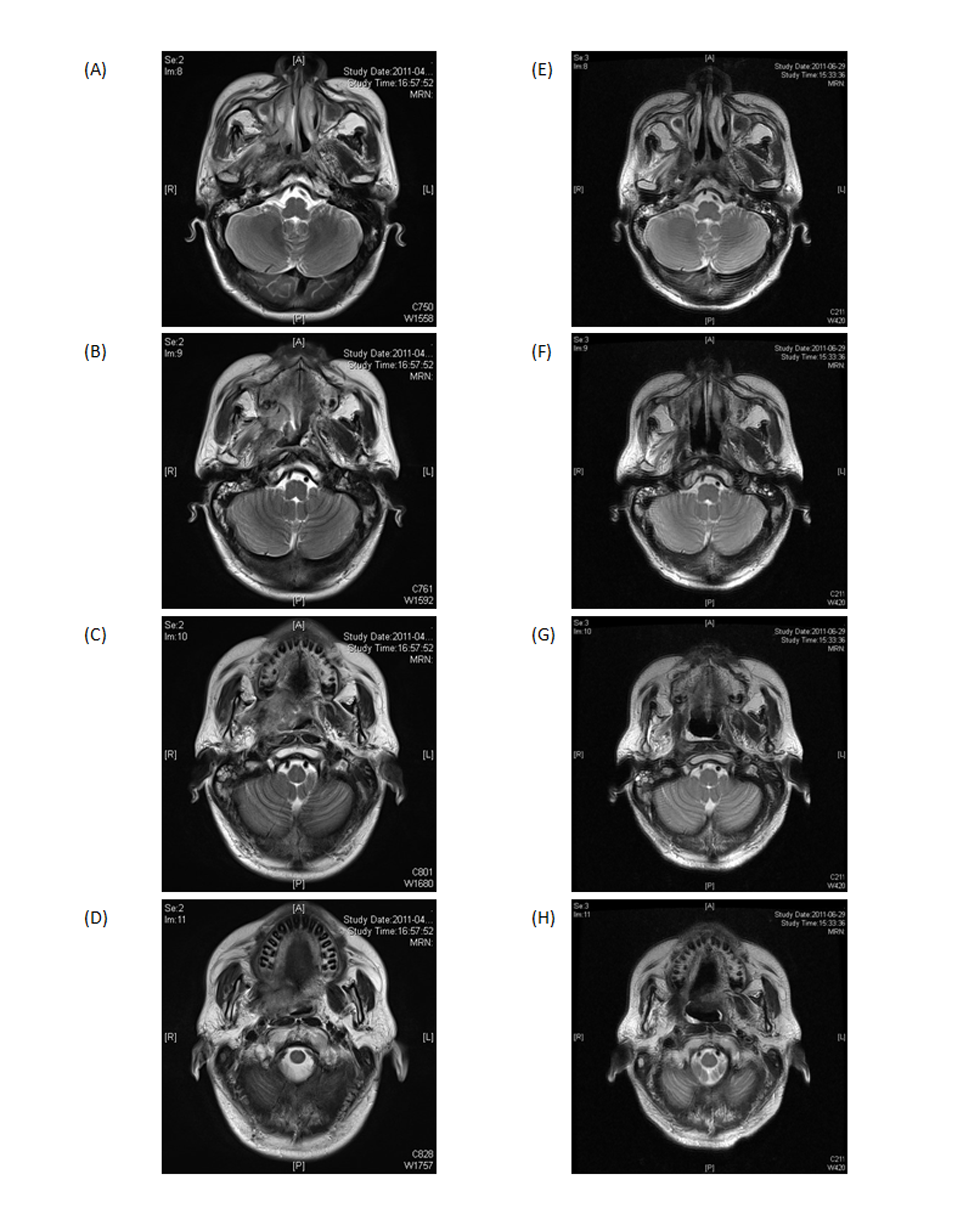

Supplement: S1 Fig — (TIF) [file pone.0125351.s001.tif]
