# Supplementary material for: Prognostic Significance of Tumor Volume in Locally Recurrent Nasopharyngeal Carcinoma Treated with Salvage Intensity-Modulated Radiotherapy
Source: PLoS One. 2015 Apr 30;10(4):e0125351. doi: 10.1371/journal.pone.0125351 (PMC4416016; doi:10.1371/journal.pone.0125351)
Supplement: S1 Table — (DOCX) [file pone.0125351.s002.docx]

**Table S2. Major late toxicities of patients with different initial radiation dose**

|  | Toxicity | 1^st^ course RT dose ≤70Gy (N=179) | 1^st^ course RT dose>70Gy (N=112) | ALL (N=291) |
| --- | --- | --- | --- | --- |
| Before 2^nd^ course RT | ulcer or necrosis of nasopharyngeal mucosa | 16 (8.9%) | 15 (13.4%) | 31 (10.7%) |
|  | trismus | 15 (8.4%) | 9 (8.0%) | 24 (8.2%) |
|  | temporal lobe necrosis | 7 (3.9%) | 6 (5.4%) | 13 (4.5%) |
|  | hearing deficit | 5 (2.8%) | 4 (3.6%) | 9 (3.1%) |
|  | cranial nerve palsy | 7 (3.9%) | 2 (1.8%) | 9 (3.1%) |
|  | vision deficit | 2 (1.1%) | 1 (0.9%) | 2 (0.7%) |
| After 2^nd^ course RT | ulcer or necrosis of the nasopharyngeal mucosa | 57 (31.8%) | 41 (36.6%) | 98 (33.7%) |
|  | trismus | 53 (29.6%) | 35 (31.2%) | 88 (30.2%) |
|  | temporal lobe necrosis | 54 (30.2%) | 24 (21.4%) | 78 (30%) |
|  | massive hemorrhage | 28 (15.6%) | 22 (19.6%) | 50 (17.2%) |
|  | hearing deficit | 45 (25.1%) | 25 (22.3%) | 70 (24.1%) |
|  | severe headache | 36 (20.1%) | 20 (17.9%) | 56 (19.2%) |
|  | difficulty in feeding | 11 (6.2%) | 5 (4.5%) | 16 (5.5%) |
|  | difficulty in speaking | 8 (4.5%) | 7 (6.3%) | 15 (5.1%) |
|  | vision deficit | 10 (5.6%) | 3 (2.7%) | 13 (4.5%) |

Abbreviations: RT, radiotherapy
